# Bone marrow basophils provide survival signals to immature B cells *in vitro* but are dispensable *in vivo*

**DOI:** 10.1371/journal.pone.0185509

**Published:** 2017-09-28

**Authors:** Joshua M. Moreau, Selena Cen, Alexandra Berger, Caren Furlonger, Christopher J. Paige

**Affiliations:** 1 Princess Margaret Cancer Centre, University Health Network, Toronto, Canada; 2 Department of Immunology, University of Toronto, Toronto, Canada; 3 Department of Medical Biophysics, University of Toronto, Toronto, Canada; St. Vincent's Institute, AUSTRALIA

## Abstract

Immature B cells are the first B cell progenitors to express a fully formed B cell receptor and are therefore subject to extensive selection processes that act to mitigate the emergence of autoreactive clones. While it is well appreciated that most B cell generation in the bone marrow is highly dependent on access to molecules present in the local milieu, the existence of extrinsically provided factors that modulate immature B cell biology is ambiguous. Nonetheless, a population of CD49b^+^CD90^lo^ cells has demonstrated *in vitro* potential to promote immature B cell survival. Using a mouse basophil reporter strain we confirmed the identity of these CD49b^+^CD90^lo^ supportive cells as basophils. However, analysis of bone marrow B cell populations following lineage specific basophil depletion demonstrates that basophils do not have a significant role *in vivo* in modulating immature B cell biology during steady-state conditions.

## Introduction

The bone marrow microenvironment is critical in supporting B lymphopoiesis and mature B cell function. Several defined cellular niches have been identified in this organ corresponding to the localization pattern of marrow B cells and progenitors [1–3]. Moreover, the cells comprising these niches express various molecules, such as IL-7, CXCL12, and MIF, conducive to B cell survival or differentiation [1,2,4,5]. While immature B cells are found enriched within and around the bone marrow sinusoids, a definitive cellular niche supportive of their biology has not been characterized *in vivo* [6,7]. This issue is of particular significance because it is at the immature stage that central tolerance is enforced though negative selection of autoreactive B cell receptors (BCR) [8]. Maturing B cells expressing an autoreactive BCR are able to re-express the recombinase genes *RAG1* and *RAG2*; in doing so such cells have the opportunity to undergo a secondary rearrangement process termed receptor editing [9–11]. B cells successfully producing a non autroreactive BCR at this juncture are able to mature normally while unsuccessful cells are deleted and unable to contribute to the repertoire. Thus any cellular and molecular factors comprising a niche specific to immature B cells would have the potential to act as regulators of receptor editing and thereby contribute to the peripheral repertoire [8,11].

While a specific bone marrow niche for immature B cells has not been identified, a few lines of evidence hint to the existence of such a feature and its potential importance to central tolerance. Immature B cells from mice expressing a transgenic autoreactive BCR had low expression levels of BAFF receptor while non autoreactive cells maintained receptor expression levels sufficient to induce signaling [12]. Moreover, BAFF was found to aid the generation of CD23^+^ transitional cells from non autoreactive immature cells, but not autoreactive one [12]. This is further substantiated by experiments demonstrating that IL-4 works synergistically with BAFF to promote immature B cell maturation into CD23^+^ transitional cells [13]. Other experiments have indicated that autoreactive and non-autoreactive immature B cells differentially localize within the marrow dictated by responsiveness to the chemoattractant S1P [14]. It has also been observed that co-culturing immature B cells with bone marrow cells provided protection against anti-BCR induced apoptosis and enhanced *RAG* expression [15,16]. This response involved contact dependent signals and was narrowed down to a non-lymphocyte cellular fraction contained within the CD90^lo^CD49b^+^ flow cytometry gate [15,16]. Subsequent work has noted the similar phenotype of these cells to basophils, including expression of CD90, CD49b, and asialo-GM1 [17].

As basophils are known to express high levels of both BAFF and IL-4, have been shown to support plasma cell survival, and exhibit a cell surface phenotype consistent with a CD90^lo^CD49b^+^ cell population we hypothesized that this cell type comprises part of the immature B cell niche *in vivo* [17–21]. Using Basoph8 lineage specific reporter mice we demonstrate that the *in vitro* effect of bone marrow CD90^lo^CD49b^+^ cells on B cells is indeed attributable to basophils [22]. However, lineage specific ablation of basophils by crossing Basoph8 mice to ROSA-DTA mice failed to yield any obvious abnormalities in B cell development or receptor editing. Thus our data indicates that while basophils are capable of supporting B cell survival they are expendable for modifying immature B cell biology *in vivo*.

## Materials and methods

### Mice

Basoph8 (C.129S4(B6)-*Mcpt8*^*tm1(cre)Lky*^/J) and ROSA-DTA (C.129P2(B6)-*Gt(ROSA)26Sor*^*tm1(DTA)Lky*^/J) mice were purchased from The Jackson Laboratory. Mice were housed and bred under specific pathogen-free conditions in the animal facilities of the Princess Margaret Cancer Centre, University Health Network (UHN). Experiments were performed on 7-20 week old male and female mice (as indicated) according to protocols approved by the UHN Animal Care Committee. Euthanasia was carried out by isoflurane overdose followed with cervical dislocation. Individual experiments were always sex and age matched and compared littermates. *In vivo* sinusoidal labeling was accomplished by IV injection of 1 μg Armenian hamster anti-mouse FcɛRIα (MAR-1; Biolegend) or rat anti-mouse B220 (RA3-6B2; eBioscience) 2 minutes prior to euthanasia.

### Cell isolation and flow cytometry

Bone marrow single-cell suspensions were made by flushing femurs and tibiae with PBS + 2% fetal calf serum (FCS). All cell suspensions were treated with ACK buffer for red cell lysis. For flow cytometic analysis cell suspensions were stained with the appropriate combination of the following antibodies: anti-FceRI-PE (MAR-1; BioLegend); ant-CD49b-PE-Cy7 (DX5; Biolegend); anti-CD90.2-APC (30-H12; Biolegend); anti-CD19-APC (1D3; eBioscience); anti-IgM-PE-Cy7 (RMM-1; Biolegend); anti-IgD-eFluor450 (11-26; eBioscience); anti-CD93-PE (AA4.1; Biolegend); anti-CD2-FITC (RM2-5; BD Biosciences). Dead cells were excluded with Zombie UV Fixable viability dye (BioLegend). For cell cycle analysis and Nicoletti assay cells were fixed with the FOXP3/Transcription Factor Staining Buffer Set (eBioscience) and DNA was stained with 4,6 diamidino-2-phenylindole (DAPI; BioLegend). Flow cytometry was conducted using an LSRFortessa 5-laser (325; 405; 488; 561; 632) configuration (BD Biosciences). For FACS cells were collected using a MoFlo Astrios (Beckman Coulter) and sorted directly into Opti-MEM+ 10% FCS Media.

### Cell cultures

CD19^+^CD2^+^IgD^-^ or CD19+CD2+IgM^-^IgD^-^ cells were cultured at 5 x 10^5^ cells/mL in 96-well plates with Opti-MEM (Thermo Fisher Scientific, Waltham, USA) supplemented with 10% fetal calf serum, 100 μg/mL penicillin and streptomycin, 2.4 g/L NaHCO_3_ and 50 μM 2-Mercaptoethanol. YFP^+^CD49b^+^CD90^lo^ or YFP^-^CD49b^+^CD90^lo^ cells were added to wells at 2 x10^4^/mL, as indicated. Some wells included the addition of 20 μg/mL goat anti-mouse IgM, μ chain specific F(ab’)_2_ (Jackson ImmunoResearch Laboratories). Cultures were left overnight (approximately 18 hours) before being harvested for cell survival analysis. In experiments using CD19+CD2+IgM^-^IgD^-^ progenitors cultures were examined after two days.

### Enumeration of total organ cell numbers

To obtain organ cell counts isolated cell suspensions from a single mouse leg was diluted in Trypan Blue (Sigma) and live cells counted using a hemocytometer. The number of live cells was multiplied by 10.6 since radiographic isotype distribution studies have found that one set of mouse femur and tibia contain 9.4% of the total marrow [23].

### Real-time PCR

B cells were purified by magnetic cell selection using a mouse CD19 positive selection kit (STEMCELL Technologies). Single cell suspensions were lysed in TRIzol (ThermoFisher Scientific) and RNA extracted by phenol/chloroform ethanol precipitation. cDNA was prepared using RT^2^ First Strand Kit (Qiagen), while qPCR was performed using RT^2^ SYBR Green Mastermixes (Qiagen) both according to manufacturer protocols with a mouse specific RAG1 primers (cat. PPM24586F Qiagen) or mouse specific B-actin primers: 5’-ACGGCCAGGTCATCACTATTG-3’; 5’- CAAGAAGGAAGGCTGGAAAAGAG-3’. Samples were run on an ABI SDS 7900HT (Applied Biosystems) using a standard protocol for *RAG1* expression: 2 min at 50°C; 95°C; 40 cycles of 15 s at 95°C and 1 min at 60°C. Serial dilutions for each sample were tested for linearity in amplification. Differences in expression levels were calculated using the comparative CT method.

### Cytospins

Cytospins were made from sorted CD49b^+^CD90^lo^YFP^+^ and CD49b^+^CD90^lo^YFP^-^ populations and Giemsa (Sigma-Aldrich) stained according to manufacturer instructions. Cytospin slides were scanned at 40x magnification using an Aperio Slide Scanner (Leica Biosystems).

### Statistical analysis

Statistical analysis was conducted using GraphPad Prisim v6 (GraphPad Software). Significance levels were defined as *p<0.05; **p<0.01; ***p<0.001 as determined by one-way ANOVA with a Dunnett’s multiple comparisons test; one-way ANOVA with a Tukey’s multiple comparisons test; two-way ANOVA with a Sidak’s multiple comparisons test.

## Results

### CD49b^+^CD90^lo^ bone marrow cells are highly enriched for basophils

**Fig 1:**
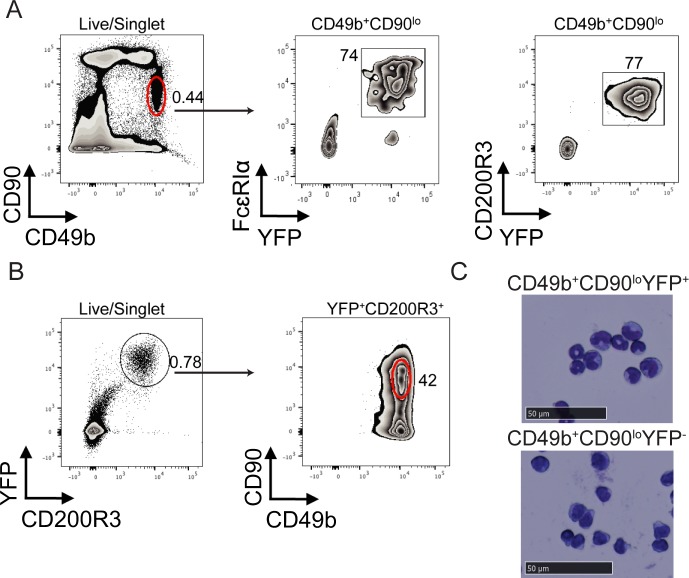
Bone marrow basophils are largely CD49b^+^CD90^lo^. (A, B) Bone marrow from 7-16 week old Basoph8 mice was examined for expression of CD49b, CD90, FcɛRIα, and YFP by flow cytometry. Representative flow plots show the frequency of YFP^+^ cells in the CD49b^+^CD90^lo^ gate (A) as well as the expression patterns of CD49b and CD90 in YFP^+^ cells (B). (C) CD49b^+^CD90^lo^YFP^+^ or CD49b^+^CD90^lo^YFP^-^ cells were FACS sorted and Giemsa stained. Representative cytospins are shown.

Previous studies have noted a bone marrow derived cell population that protected immature B cells from BCR crosslinking induced apoptosis [15,16]. These cells were found to be CD49b^+^CD90^lo^ and asialo-GM1^+^ while negative for B220, MHC class II, CD11c, CD4, CD5, and CD8 [15,16]. Further, the supportive cells could be isolated from *Rag-2*^*-/-*^ and *IL-2Rϒ*^*-/-*^ mice [16]. To test the hypothesis that these cells were basophils we obtained Basoph8 mice from the Jackson Laboratory. Basoph8 is a reporter mouse stain where the mast cell protease 8 (*Mcpt8*) gene has been replaced with a cassette containing sequences encoding YFP and humanized Cre recombinase [22]. Mcpt8 is a basophil specific protein found in mice but not humans and does not appear to be required for basophil development [22,24]. Staining bone marrow from Basoph8 mice for CD49b, CD90, CD200R3, and the high affinity IgE receptor revealed a dramatic enrichment of basophils (YFP^+^CD200R3^+^FcɛRIα^+^) in the CD49b^+^CD90^lo^ flow cytometry gate as compared to other cell populations (Fig 1A). The CD49b^+^CD90^lo^ population represented roughly half of cells doubly expressing YFP and CD200R3, while the rest were CD49b^+^CD90^-^ (Fig 1B). To further confirm the identity of the CD49b^+^CD90^lo^YFP^+^ population we FACS sorted these as well as CD49b^+^CD90^lo^YFP^-^ cells and made cytospins. Giemsa staining reveled that the YFP^+^ fraction was homogenous with most cells exhibiting bilobed nuclei and light granule staining typical of mouse basophils [25,26]. In contrast, the YFP^-^ population appeared more lymphocytic with a large nuclear to cytoplasmic ratio (Fig 1C).

### Bone marrow basophils provide *in vitro* support for immature B cell survival

**Fig 2:**
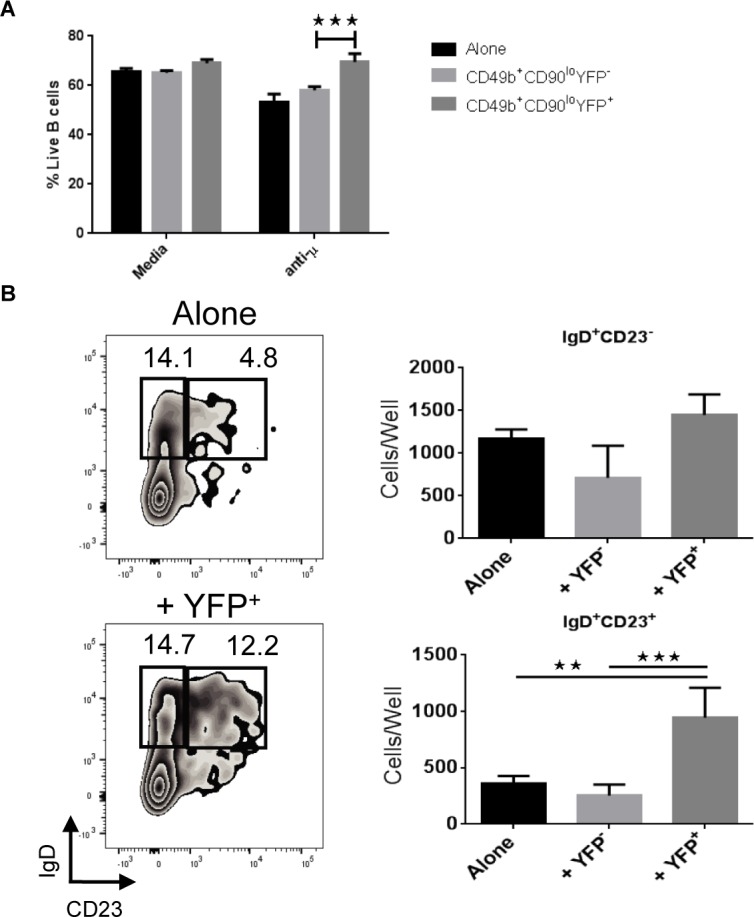
Basophils protect B cell progenitors in co-culture systems. (A) Bone marrow from 7-16 week old Basoph8 mice was stained for CD49b and CD90. CD49b^+^CD90^lo^YFP^+^ and CD49b^-^CD90^lo^YFP^-^ cells were sorted by FACS and co-cultured overnight together with CD2^+^IgD^-^ FACS sorted B cell progenitors at a ratio of 1:25. In some culture wells anti-μ was added at 20 μg/mL to stimulate BCR crosslinking. Cell survival was measured by a Nicoletti assay. (B) Sorted CD2^+^IgM^-^IgD^-^ B cell progenitors were co-cultured for two days with YFP^+^ cells. The absolute number of CD2^+^IgM^+^CD23^-^ and CD2^+^IgM^+^CD23^+^ B cells was then determined by flow cytometry. Data shown as mean ± standard deviation and are representative of three individual experiments; *p<0.05; **p<0.01; ***p<0.001.

As the CD49b^+^CD90^lo^ bone marrow cell population is heterogeneous, we sought to determine whether the basophil fraction among CD49b^+^CD90^lo^ cells was responsible for protecting B cells from BCR crosslinking induced apoptosis, *in vitro*. To accomplish this bone marrow from Basoph8 mice was collected and stained for both CD49b and CD90. CD49b^+^CD90^lo^ cells were then sorted by FACS into YFP^+^ (basophils) and YFP^-^ fractions. We co-cultured these populations overnight with CD2^+^IgD^-^ B cell progenitors at a 1:25 ratio in the presence or absence of BCR crosslinking anti-μ antibody. The frequency of live B cells was then measured with a Nicoletti assay to gate out apoptotic cells [27]. As shown in Fig 2A, co-culture with CD49b^+^CD90^lo^YFP^+^ cells reversed the decline in B cell survival seen following treatment with anti-μ. Conversely, CD49b^+^CD90^lo^YFP^-^ cells had no such influence. As an additional test of the *in vitro* capacity for basophils to support B cell progenitors CD49b^+^CD90^lo^YFP^+^ and CD49b^+^CD90^lo^YFP^-^ cells were sorted and co-cultured with CD2^+^IgM^-^IgD^-^ B cells, as before. Two days later the frequency and absolute numbers of IgD^+^CD23^-^ and IgD^+^CD23^+^ cells were assessed by flow cytometry. These markers indicate the maturation of B cell progenitors into the early transitional and late transitional/mature stages respectively [28]. The addition of YFP^+^ cells to these cultures greatly enhanced the recovery of IgD^+^CD23^+^ cells (Fig 2B). Collectively, these data indicate that basophils are in fact the CD49b^+^CD90^lo^ bone marrow cell population that provides support to developing B cells, *in vitro*.

### Basophils localize to the bone marrow sinusoids

**Fig 3:**
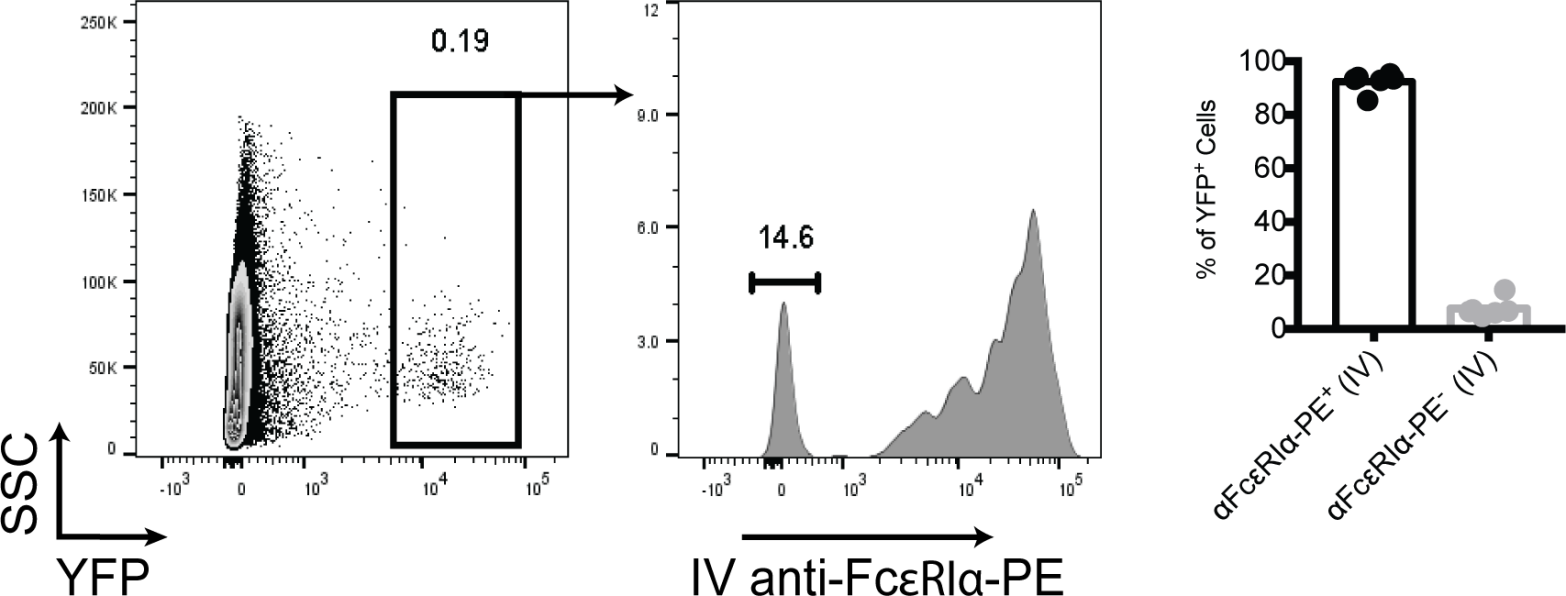
Basophil share marrow localization patterns with B cells. Basoph8 mice were intravenously injected with 1 μg anti-FcεRIα-PE and euthanized two minutes later. FcεRIα-PE staining was then assessed by flow cytometry. Shown is a representative flow plot and quantification from three independent experiments.

Having seen that basophils are capable of providing a survival advantage to immature B cells when co-cultured, we aimed to determine whether basophils provide the same support *in vivo*. Previous work has determined that CD49b^+^ cells maintain close contact with B cells in the bone marrow tissue [16]. Since immature B cells are known to be preferentially distributed near bone marrow sinusoids we examined if basophils are similarly localized near the sinusoids [4,7]. To assess this, basoph8 mice were intravenously injected with anti-FcεRIα antibody conjugated to R-phycoerythrin and euthanized two minutes later. The large molecular weight of this flurophore prevents it from rapidly diffusing into the parenchymal tissue and therefore when conjugated to a specific antibody has been shown to preferentially label sinusoidal cells [7]. A large majority of YFP^+^ bone marrow cells were found to be FcεRIα-PE^+^ indicating that like immature B cells basophils tend to accumulate in and around the marrow vasculature (Fig 3).

### Lineage specific depletion of basophils does not alter B cell development *in vivo*

**Fig 4:**
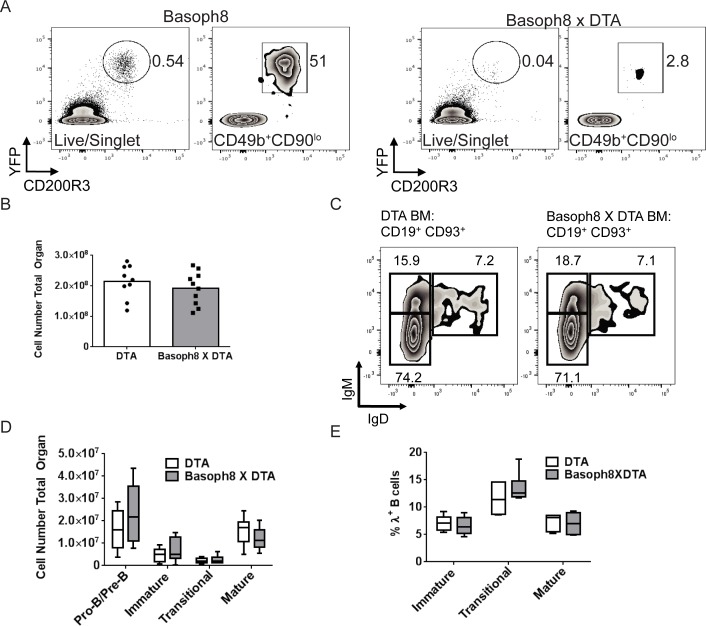
Basophils do not affect progenitor B cell numbers and λ light chain usage *in vivo*. (A-E) Basoph8 mice were crossed to the ROSA-DTA strain and bone marrow cell numbers and B cell populations were enumerated by flow cytometry. Age and sex matched ROSA-DTA mice were used as controls. (B) Total bone marrow cellularity was calculated by trypan blue staining and counting with a hemocytometer. (A, C) Representative bone marrow flow cytometry plots are shown. (D) B cell populations were calculated with pro-B and pre-B defined as CD19^+^CD93^+^IgM^-^IgD^-^, immature-B as CD19^+^CD93^+^IgM^+^IgD^-^, transitional as CD19^+^CD93^+^IgM^+^IgD^+^, and mature as CD19^+^CD93^-^IgM^+^IgD^+^. (E) Bone marrow was stained for λ light chain. Data shown (D, E) as median + quartile box-and-whisker plots and is pooled from three individual experiments using mice aged 10-16 weeks; n = 9 ROSA-DTA, n = 10 Basoph8 X DTA. All groups are non-significant.

**Fig 5:**
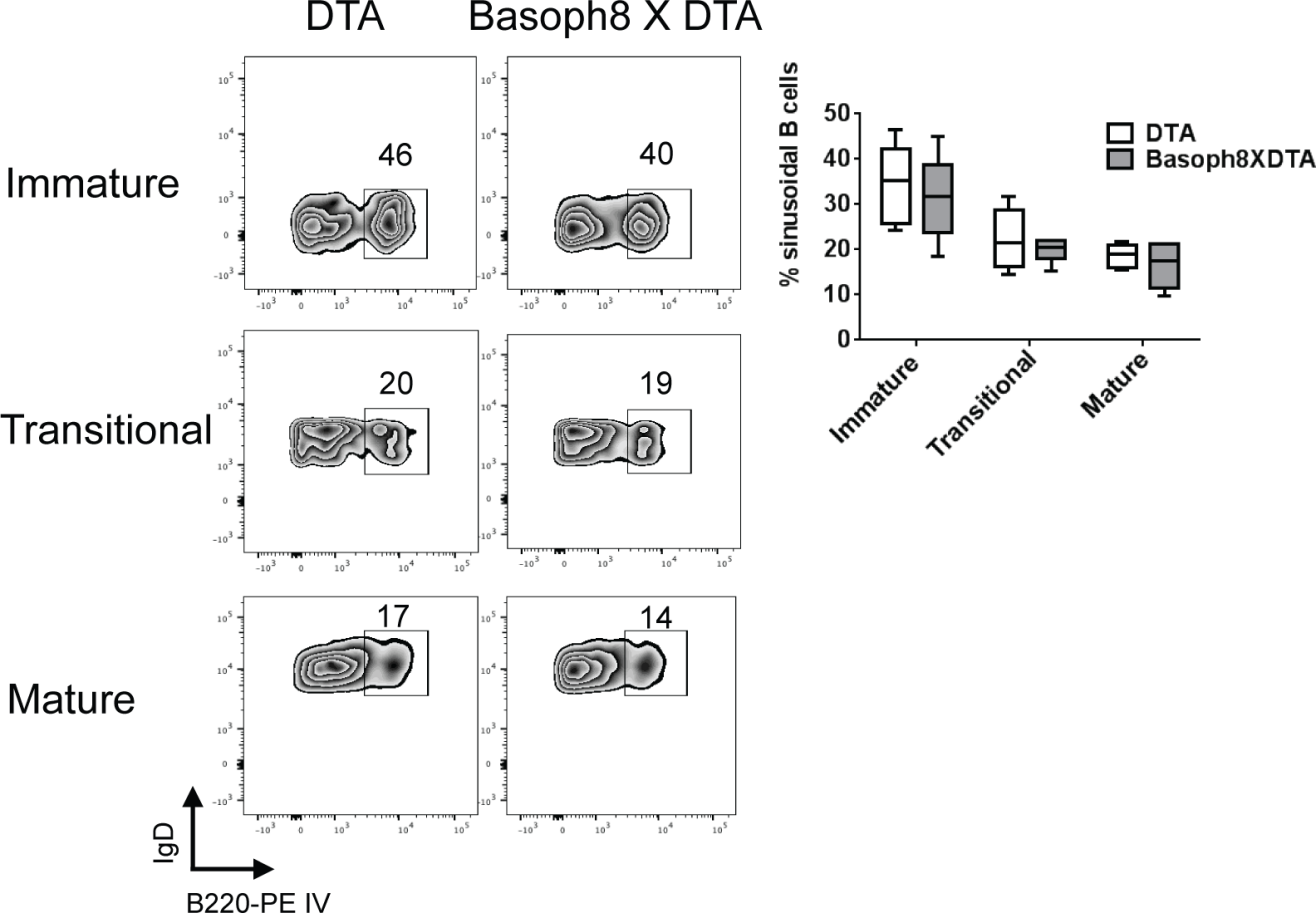
Basophils do not influence immature B cell localization. Basoph8 X ROSA-DTA mice were intravenously injected with 1 μg anti-B220 conjugated to R-phycoerythin and euthanized two minutes later. The frequency of B220-PE staining B cells was determined by flow cytometry. Left panels show representative flow plots while the right graph shows data pooled from two independent experiments in a median + quartile box-and-whisker plot; n = 6 all groups mice were 8-16 weeks of age. All groups are non-significant.

To test if basophils influence B cell development *in vivo*, we crossed Basoph8 and ROSA-DTA mouse strains and analyzed the bone marrow B cell compartment in the resulting offspring. This cross produces lineage specific expression of diphtheria toxin and therefore efficient deletion of basophils [22](Fig 4A). Surprisingly, examination of the bone marrow of Basoph8 x ROSA-DT mice did not reveal any notable anomalies despite thorough basophil deletion when compared to age and sex matched ROSA-DT controls. Total marrow cellularity, pro-B and pre-B, immature-B, transitional, and mature B cell populations were normal (Fig 4B–4D). In addition there was no difference in the usage of λ light chain among immature, transitional and mature bone marrow B cells (Fig 4E). Immature B cells, especially non autoreactive ones, are known to collect in and around the bone marrow sinusoids [6,7,14]. To determine if basophils could influence B cell localization to the sinusoids we intravenously injected mice with anti-B220 conjugated to R-phycoerythin and euthanized the mice two minutes later, as described above. Basoph8 x ROSA-DTA mice demonstrated normal distributions of B cells as compared to ROSA-DTA control animals (Fig 5).

**Fig 6:**
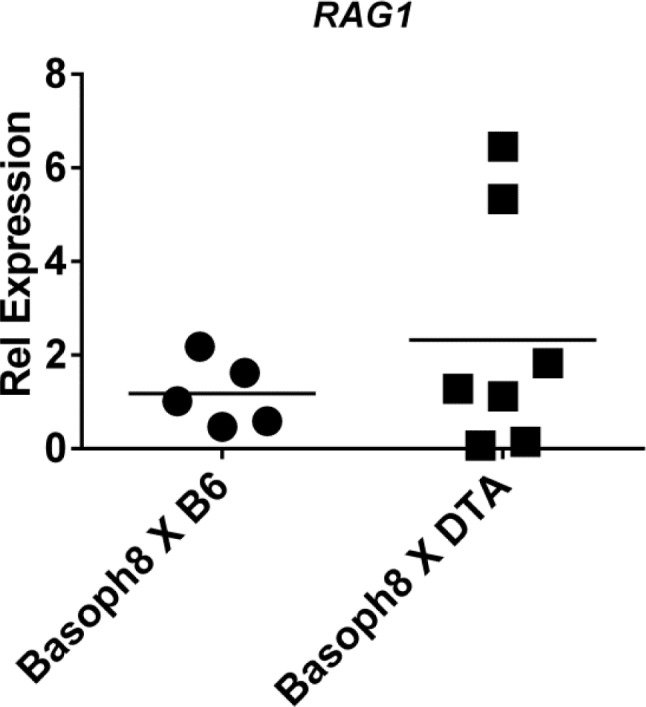
*RAG1* expression is unchanged with basophil deletion. Real-time PCR analysis of *RAG1* in B cells from Basoph8 X ROSA-DTA or Basoph8 X B6 mice. B cells were purified by magnetic cell selection before RNA extraction. *RAG1* levels were first normalized to *Beta-actin* and then to mean *RAG1* levels in Basoph8 X B6 (control) samples. Data are from three independent experiments in which B cells from mice aged 12-20 weeks were pooled from one to three mice.

A key finding of earlier work examining the potential for CD49b^+^CD90^lo^ cells to support immature B cells was that when these cells were co-cultured together and treated with an anti-IgM BCR crossing antibody B cells were induced to express *RAG* genes [15]. We sought to determine if basophil deletion would mirror these *in vitro* experiments and produce a reduction of B cell *RAG* expression. To test this we measured *RAG1* expression by real-time PCR in purified B cells derived from Basoph8 x ROSA-DT or Basoph8 X B6 mice. No significant difference in expression level was discernable (Fig 6). Overall, these analyses indicate that lineage specific deletion of basophils does not have a significant impact on the normal development of B cells *in vivo*.

## Discussion

Immature and transitional B cells represent a crucial stage in B lineage development [29]. These are the first B cell progenitors to express a fully formed, lineage defining BCR; nonetheless, by virtue of the necessity to regulate autoreactive potential within the mature B cell repertoire they are highly sensitive to selection processes. Crosslinking of immature and transitional BCRs by self-antigen is likely to induce receptor editing, deletion, or anergy [8,10,29]. Passing such selection events promotes survival, further differentiation, and relocation to mature immune environments [1,8]. An intriguing possibility is that cell extrinsic signals present in the local environment in which an immature B cell encounters antigen could act to modify the selection processes of immature and transitional cells [8,11]. By extension, cellular or molecular factors governing this stage of B cell development would be positioned to influence the mature BCR repertoire and therefore peripheral B cell function.

In the current study, we sought to examine the hypothesis that bone marrow basophils contribute to supporting immature and transitional B cell development. Previous work research demonstrated that a peculiar CD49b^+^CD90^lo^ bone marrow cellular fraction is capable of promoting the survival of immature and transitional B cells during exposure to BCR crosslinking [15,16]. Using Basoph8 lineage reporter mice we have unambiguously identified these cells as basopholis and validated that they are capable of providing B cells beneficial signals during *in vitro* co-culture. However, when we crossed Basoph8 with ROSA-DTA mice so as to generate basophil ablated mice we could find no defects in the populations numbers of bone marrow B cells, the usage of the λ light chain, or the accumulation of B cells within the marrow sinusoids. In addition, there was no difference in the expression level of *RAG1* in basophil deleted mice. We interpret these findings to imply that while basophils possess the molecular machinery to influence immature and transitional B cells, they are dispensable or redundant *in vivo*.

Given that we have previously documented an accumulation of CD49b^+^CD90^lo^ cells in the bone marrow in response to adjuvant induced inflammation it tempting to speculate that the basophils may gain functional relevance to B cells during non steady-state conditions such infection [30]. Potentially, *in vitro* culture conditions are relatively skewed towards an environment conducive to basophil help based upon the balance of various nutrients, inflammatory factors, and cellular stress. The feasibility of this idea is supported by a discrepancy seen between this and previous work: when CD49b^+^CD90^lo^ cells were targeted for deletion by repetitive administration of antibodies against asialo-GM1 a concurrent loss of immature B cells was observed [16]. While this result may have been due to off target effects of the anti-asialo-GM1, possibly the punctuated apoptosis of marrow resident cells induced by this treatment may have created an environment where immature B cells were dependent on basophil availability.

Through the experiments presented we have failed to find evidence that implicates basophils as major regulators of immature B cell biology during steady-state marrow conditions. However, it is clear that in the conditions of *in vitro* co-culture basophils efficiently support B cell survival and possibly maturation. In this, regard our experiments are in agreement with previous studies as well as the profile of basophil as IL-4^+^BAFF^+^CD40L^+^ cells with a known propensity to support B cells in a variety of contexts [18,19,21]. Since our main aim was to determine if there is a role for basophils *in vivo*, we did not explore the extent to which any of these or other cell supplied molecules may contribute to B cell survival. Deeper investigation of basophil-B cell behavior during co-culture has potential to serve as a useful vehicle for the discovery of B cell modulating factors. Similarly, the very fact that basophils enhance B cell survival *in vitro* but not *in* vivo bolsters the initial rational for our study: environment matters in B cell biology.
